# Marine Cyclic Guanidine Alkaloids Monanchomycalin B and Urupocidin A Act as Inhibitors of TRPV1, TRPV2 and TRPV3, but not TRPA1 Receptors

**DOI:** 10.3390/md15040087

**Published:** 2017-03-23

**Authors:** Yuliya Korolkova, Tatyana Makarieva, Kseniya Tabakmakher, Larisa Shubina, Ekaterina Kudryashova, Yaroslav Andreev, Irina Mosharova, Hyi-Seung Lee, Yeon-Ju Lee, Sergey Kozlov

**Affiliations:** 1 Shemyakin-Ovchinnikov Institute of Bioorganic Chemistry, Russian Academy of Sciences, Miklukho-Maklaya Street, 16/10, Moscow 117997, Russia; ay@land.ru (Y.A.); mosharova@mail.ru (I.M.); serg@ibch.ru (S.K.); 2 G.B. Elyakov Pacific Institute of Bioorganic Chemistry (PIBOC), Russian Academy of Sciences, Prospect 100 let Vladivostoku, 159, Vladivostok 690022, Russia; makarieva@piboc.dvo.ru (T.M.); dark_xen@mail.ru (K.T.); shubina@piboc.dvo.ru (L.S.); catrinog.81@mail.ru (E.K.); 3 Sechenov First Moscow State Medical University, Institute of Molecular Medicine, Trubetskaya str.8, bld. 2, Moscow 119991, Russia; 4 Korea Institute of Ocean Science & Technology, Marine Natural Products Laboratory, Ansan 426-744, Korea; hslee@kiost.ac.kr (H.-S.L.); yjlee@kiost.ac.kr (Y.-J.L.)

**Keywords:** guanidine alkaloids, sponge, *Monanchora pulchra*, TRPV1, TRPV2, TRPV3, TRPA1

## Abstract

Marine sponges contain a variety of low-molecular-weight compounds including guanidine alkaloids possessing different biological activities. Monanchomycalin B and urupocidin A were isolated from the marine sponge *Monanchora pulchra.* We found that they act as inhibitors of the TRPV1, TRPV2, and TRPV3 channels, but are inactive against the TRPA1 receptor. Monanchomycalin B is the most active among all published marine alkaloids (EC_50_ 6.02, 2.84, and 3.25 μM for TRPV1, TRPV2, and TRPV3, correspondingly). Moreover, monanchomycalin B and urupocidin A are the first samples of marine alkaloids affecting the TRPV2 receptor. Two semi-synthetic urupocidin A derivatives were also obtained and tested against TRP (Transient Receptor Potential) receptors that allowed us to collect some data concerning the structure-activity relationship in this series of compounds. We showed that the removal of one of three side chains or double bonds in the other side chains in urupocidin A led to a decrease of the inhibitory activities. New ligands specific to the TRPV subfamily may be useful for the design of medicines as in the study of TRP channels biology.

## 1. Introduction

Biologically active cyclic guanidine alkaloids are known to be characteristic metabolites of tropical marine sponges belonging to the genera *Ptilocaulis*, *Hemimycale*, *Crambe*, *Batzella*, *Clathria*, and *Monanchora* [1,2]. In recent years it has been shown that the far-eastern marine sponge *Monanchora pulchra* is also a rich source of novel pentacyclic [3,4,5,6,7], bicyclic [8] and acyclic guanidine alkaloids [9,10]. It is interesting that alkaloids produced in the sponges from different locations and depths had a variety of structures. The reasons for these differences are not clear. The alkaloids from the sponge *M. pulchra* demonstrate a broad spectrum of biological activities, including potent cytotoxic effects [4,5,6], induction of cellular autophagy and lysosomal membrane permeabilization [11], apoptosis [3], as well as inhibition of some cellular receptors [9,10,12,13]. For instance, it was shown earlier that the pentacyclic guanidine alkaloid monanchocidin A demonstrated inhibitory activities in electrophysiology experiments on the mouse muscle and rat α7 nAChRs [13], while acyclic guanidine alkaloids pulchranins A–C inhibited the TRPV1 receptor [9,10,12].

The main direction of our research is the isolation of new natural products from marine life, and investigation and characterization of their biological activity and molecular targets as well as possible therapeutic applications.

Transient receptor potential (TRP) receptors are emerging targets that have attracted pharmaceutical interest. Twenty-eight mammalian TRP receptors are known and may be grouped into six subfamilies: TRPC (“Canonical”), TRPA (“Ankyrin”), TRPV (“Vanilloid”), TRPM (“Melastatin”), TRPP (“Polycystin”), TRPML (“Mucolipin”) [14]. TRP receptors were shown to be ubiquitous in the human organism and they are expressed in many tissues, where they regulate different cell functions and are implicated in the pathogenesis of various acquired and inherited human diseases [14,15,16]. Surprisingly, only four of 28 mammalian TRP (namely TRPV1, TRPV3, TRPA1, TRPM8) counterparts have yielded clinical stage ligands [17], despite their relevance to a multitude of pathologies.

To date, members of the TRPV and TRPA subfamilies have been implicated in the sensory detection transduction of nociception and pain [17,18,19]. A few neuropathologies connected with the deregulation of these receptors have been identified. Potent antagonists of TRPV1, TRPV3 and TRPA1 have been advanced into clinical trials for the treatment of inflammatory, neuropathic and visceral pain as analgesic agents [17]. Moreover, TRPV2 and TRPA1 seem to be involved in insulin secretion [20], TRPV1 and TRPV2 in heart hypertrophy [14,15], TRPV3 in skin disorders [14], TRPV1 and TRPA1 in airway irritation and cough [21], and TRPV1, TRPV2 and TRPA1 in cancer [22,23]. Thus, a better understanding of the TRP channel biology promises a new opportunity for developing of innovative medications.

TRP channels are primary targets for a number of natural products [24]. Herein we report the isolation of known pentacyclic (monanchomycalin B) and bicyclic (urupocidin A) guanidine alkaloids from two new collections of the sponge *M. pulchra* as well as the production of two semi-synthetic derivatives of urupocidin A and the characterization of their activities against the rat TRPV1 (rTRPV1), mouse TRPV2 (mTRPV2), human TRPV3 (hTRPV3) and rat TRPA1 (rTRPA1) channels. The usefulness of new TRPV ligands for the design of medicines and in the study of TRP channels biology is discussed.

## 2. Results and Discussion

### 2.1. Isolation and Stucture of Individual Compounds

**Figure 1:**
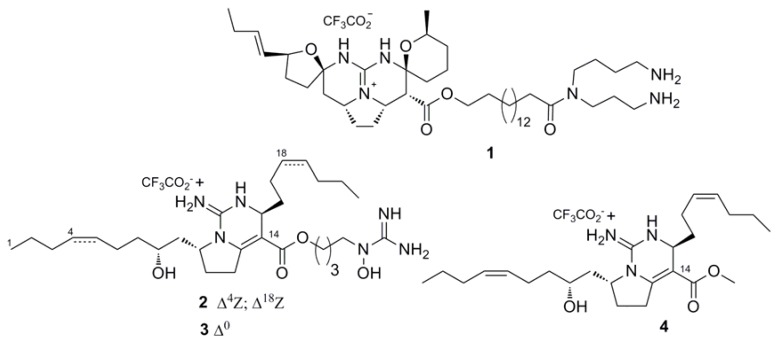
Structures of natural compounds **1**, **2** and semi-synthetic compounds **3**, **4**.

The samples of the marine sponge *M. pulchra* were collected in Okhotsk Sea (Kuril Islands region). The EtOH extract of sample N 047-243 of the sponge *M. pulchra* was concentrated. The ethanol-soluble materials were further subjected to flash column chromatography on YMC*GEL ODS-A reversed-phase sorbent to obtain a mixture of guanidine alkaloids. Separation of the mixture and purification were carried out by repeated HPLC to provide pure monanchomycalin B (**1**) (Figure 1). The structure of the compound was assigned through comparison of their spectral data with those reported in [5]. The EtOH extract of sample N 043-583 of the sponge *M. pulchra* was concentrated and partitioned between H_2_O and *n*-BuOH. After evaporation of the solvent, the BuOH-soluble materials were partitioned between *n*-hexane and aqueous EtOH. The ethanol-soluble materials were further subjected to repeated column chromatography on Sephadex LH-20 to obtain a mixture of guanidine alkaloids. Separation of the mixture and purification were carried out by repeated HPLC to provide pure urupocidin A (**2**) (Figure 1). The structure of the compound was assigned through comparison of its spectral data with those reported earlier [8]. The semi-synthetic derivatives (**3**) and (**4**) (Figure 1) were obtained from urupocidin A by an alkaline hydrolysis or a hydrogenation as described earlier, and structures of these compounds were assigned through comparison of their spectral data with those reported earlier at the structure elucidation of urupocidin A [8].

Structurally, monanchomycalin B (**1**) contains the same pentacyclic core as monanchocidin A [3], but a hydrophobic moiety with spermidine residue as previously found in ptilomycalin A [25]. The urupocidin A (**2**) possesses a trisubstituted hydrocarbon chain bicyclic system with three different alkyl substituents. Tetrahydrourupocidin A (**3**) retains three alkyl fragments, but has additional hydrophobicity from two of them in comparison with **2**. The degraded product (**4**) has only two hydrophobic chains and an absence of the guanidine group in one of them when compared with **2** and **3**.

### 2.2. Functional Activity Study on TRP Receptors

**Figure 2:**
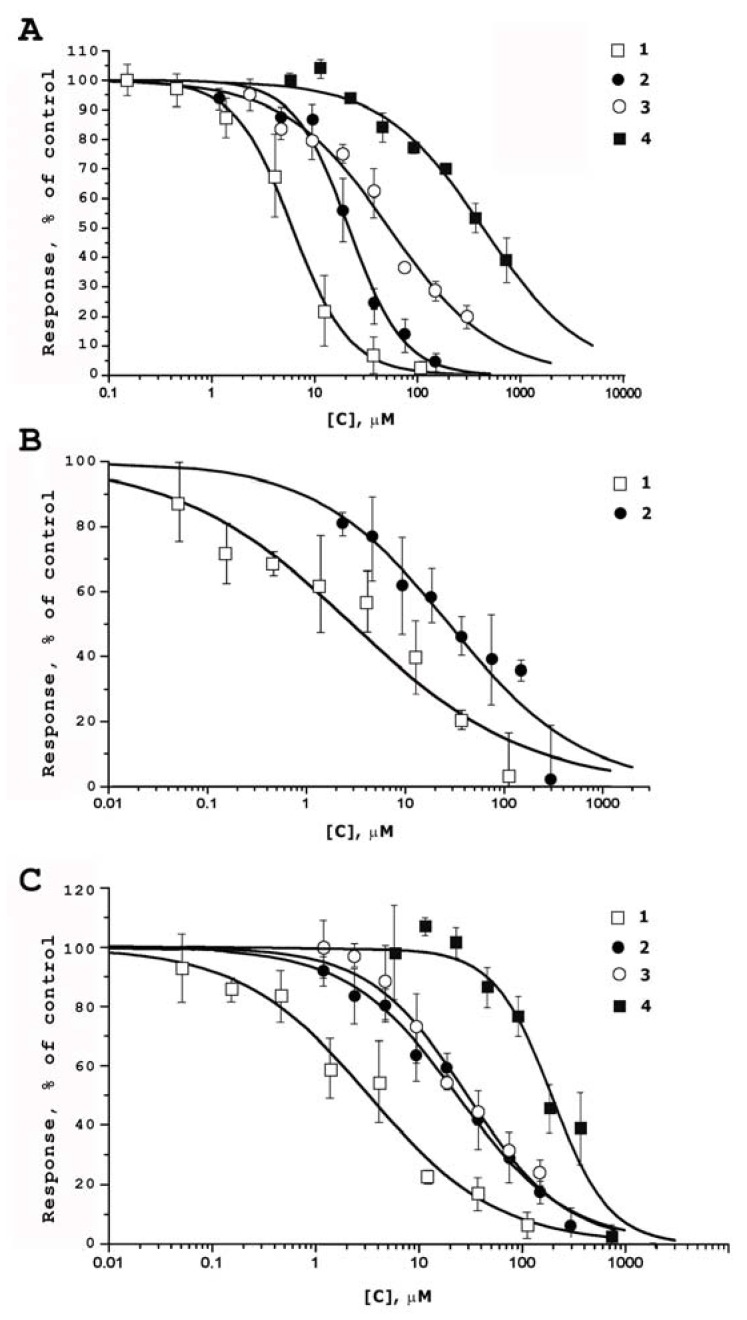
Dose-response curves for monanchomycalin B (**1**), urupocidin A (**2**), semi-synthetic urupocidin A derivatives (**3**, **4**) inhibitory activity on agonist-induced [Ca^2+^] responses in CHO cells expressing rTRPV1 (**A**); mTRPV2 (**B**); hTRPV3 (**C**). Responses were measured as the pseudo-ratio (ΔFI/FI) determined using the following formula ΔFI/FI = (FI-FIbase)/FIbase, where FI is the measured peak fluorescence intensity, Fbase is the fluorescence intensity in non-stimulated cells. Inhibitory activity was expressed as the percentage of the response in the control experiment (untreated by alkaloids cells). Data are expressed as mean ± s.e. (*n* = 4–8).

The guanidine alkaloids (**1**–**4**) were tested on inhibitory activities against rTRPV1 (Figure 2A), mTRPV2 (Figure 2B), hTRPV3 (Figure 2C) and rTRPA1 receptors expressed in CHO (Chinese hamster ovary) cells in a Fluo-4–based intracellular calcium assay [26]. The results of the study are given in Table 1.

The effect of **1**–**4** on all the TRP-expressing cells was measured after preincubation at 37 °C for 30 min for standardization. Compounds **1**–**4** showed a similar affinity without additional preincubation (data not shown). Compounds **1**–**4** did not affect non-transfected CHO cells. Capsazepine (CZP) or non-specific blocker ruthenium red (RR) were used as a positive control for TRPV1 and for the other studied TRP receptors correspondingly. A 10 µM CZP or 20 µM RR application resulted in full inhibition of the [Ca^2+^] response induced by specific agonists in TRP-expressed cells (data not shown).

Table 1 shows that the pentacyclic guanidine alkaloid monanchomycalin B (**1**) and bicyclic guanidine compounds (**2**, **3**) act as inhibitors of rTRPV1, mTRPV2 (except not tested with compound **3**), and hTRPV3 receptors. Alkaloids (**1**–**4**) did not show any activity against the rTRPA1 receptor in the whole range of investigated concentrations (1–300 µM).

Alkaloid **1** in these series of compounds possessed maximal activity on rTRPV1, mTRPV2, and hTRPV3 receptors with EC_50_ at 6.02, 2.84, and 3.25 µM, when compared with the inhibitory activities of urupocidin A (**2**) (EC_50_ at 21.47, 28.06, and 23.55 µM, respectively) and other studied compounds.

To date, only a few marine natural products have been reported as modulators of TRP receptors [9,10,12,27]. The most active of the published marine alkaloids are haliclonadiamine, the pentacyclic alkaloid from the tropical sponge *Haliclona* (*Chalinidae*) [27], and pulchranin A, the acyclic guanidine alkaloid from the far-eastern sponge *M. pulchra* [9]. Their inhibitory activity against rTRPV1 determined in the same test system was similar to that of urupocidin A (**2**) (EC_50_ 19.90 and 27.49 µM for haliclonadiamine and pulchranin A, respectively). Moreover, haliclonadiamine and pulchranin A were about four times less active toward hTRPV3 (EC_50_ 76.97 and 71.78 µM respectively). Thus, monanchomycalin B (**1**), to date, is the most active non-peptide antagonist of TRPV(1–3) receptors of marine origin.

Neither monanchomycalin B (**1**) nor urupocidin A (**2**) and its derivatives (**3** and **4**) possessed activity on rTRPA1 in contrast to the previously described marine alkaloids which did not have such selectivity of action. Earlier we showed that haliclonadiamine and pulchnanin A moderately inhibited rTRPA1 (EC_50_ 86.69 and 174.25 µM, respectively) [27].

The affinity of monanchomycalin B (**1**) and urupocidin A (**2**) to different TRPV channels changed insignificantly, which can be explained by the common mechanism of action on TRPV1, TRPV2 and TRPV3 channels and the interaction with similar structural motives on the channel surface which must, in turn, differ from TRPA1.

TRPV1, TRPV2 and TRPV3 belong to the “vanilloid” subfamily of TRP receptors and have about 40%–50% identity of amino acid sequences. TRPA1 is a single member of the “ankyrin” subfamily of TRP receptors. TRPA1 is unusual among mammalian TRP channels in that it has a very long ankyrin repeat within the *N*-terminal domain (14–18 ankyrin repeats depending on species). TRPV channels also have *N*-terminal ankyrin repeats, although they are much shorter (six repeats). It was supposed that the *N*-terminal ankyrin domain is an integration site for multiple physiological signals and specified sensitivity to thermal and chemical stimuli [28].

However, among all the TRP subfamilies, TRPA1 is the closest to TRPVs phylogenetically and functionally. TRPV1, TRPV2, TRPV3 are Ca^2+^-permeable, non-selective cation channels that can be activated by heating in heterologous expression systems (>43 °C for TRPV1, >52 °C for TRPV2, >34 °C for TRPV3). Moreover, TRPV1 is involved in the regulation of body temperature. TRPA1 is permeable to both monovalent and divalent cations [14,15,16]. TRPA1 also belongs to thermosensitive channels but TRPA1 activation by temperature is species-specific: while primate TRPA1 (macaque and human) can be activated at noxious cold temperatures (<17 °C), rodent TRPA1 channels are insensitive to cold temperatures [18]. TRPA1 is expressed in sensory neurons and co-localized with pain markers such as TRPV1 and involved in pain sensation and inflammation development [15].

Though a large number of TRPV subfamily agonists have been identified, 2-aminoethoxydiphenylborate (2-APB) was identified as a common agonist for TRPV1, TRPV2, and TRPV3 [29]. TRPA1 is activated by allyl isothiocyanate (AITC), pungent organosulfur compounds from garlic and onion (e.g., allicin and diallyl disulfide), lidocaine and some other activators of TRPV1 [30,31,32], and by carvacrol and thymol—known agonists of TRPV3 [33]. Thus, some structural and functional similarity of TRPV(i) and TRPA1 receptors may explain the non-specific action of many known ligands. The detected specificity of monanchomycalin B (**1**) and urupocidin A (**2**) to TRPV(1–3) is the important factor for the study of these receptors.

As was mentioned, TRPA1 homologues of different origins have some features in structure [28], thermosensation [18] and sensitivity to antagonists/agonists [34]. So we do not exclude that alkaloids (**1**–**4**) may affect TRPA1 of the other species. Furthermore, species-specific pharmacological properties of TRPV(1–3) as well as the activation method (pH, thermoactivation, activation by different agonists) will determine the affinity of the tested compounds (**1**–**4**).

The structure-activity relationship in urupocidin A and its semi-synthetic derivatives series (**2**–**4**) showed that natural compound (**2**) is more active then tetrahydrourupocidin (**3**) (Table 1, Figure 2). Moreover, the bicyclic derivative **4** without the hydroxyguanidine-containing substituent is almost not active against rTRPV1 and hTRPV3 receptors (Table 1). On the basis of these results, we propose that all three side chains attached to the bicyclic core of urupocidin A play important roles in inhibitory activity on TRPV1 receptors as well as on TRPV3 channels. This opens some perspective for further syntheses and searches for new guanidine inhibitors, acting on TRP receptors in this series of compounds.

TRPV1 remains the most studied receptor within the TRP channel family and has a central role in thermosensation and pain perception [18,19]. Despite a moderate homology of 43%, TRPV3 and TRPV1 show distinct tissue expression (though both are expressed in nociceptive sensory neurons), and electrophysiological and pharmacological properties [14,15]. TRPV3 is primarily localized in skin keratinocytes and also detected in other epithelia in the tongue, palate, nose, hair follicle, and distal colon. Multiple classes of small molecules are described as antagonists of TRPV1 and TRPV3 but most of them are non-selective. These molecules were reported to be effective in suppressing inflammatory and neuropathic pain in animal models and many of them have been advanced to clinical studies [17]. However, most TRPV1 antagonists caused marked hyperthermia as a side effect, prompting their withdrawal from the clinical trials. Thus, new selective and potent compounds modulating TRPV1 and TRPV3 could be extremely valuable for the design of medicines and in advancing our understanding of the TRP channels’ biology.

It is worth noting that **1** and **2** are the first marine alkaloids affecting TRPV2 receptors. In opposition to TRPV1, the osmo- and mechano-sensory–related TRPV2 is one of the least studied “vanilloid” receptors. The absence of specific TRPV2-modulating chemical tools has been causative for the current lack of knowledge on the underlying pharmacology. No selective natural TRPV2 antagonists have been validated thus far. Until now, only a few non-specific low-molecular-weight inhibitors of TRPV2 were known [23]: (a) SKF96365 is an inhibitor of non-selective cation channels and also inhibits TRPV2 activated by 2-APB; (b) ruthenium red inhibits a large number of ion channels including TRP channels and blocks TRPV2 as well; (c) tranilast, in addition to TRPV2, inhibits IgE-mediated receptor and PDGF-induced calcium entry.

The expression of TRPV2 is high in some types of cells including neurons, neuroendocrine cells, immune cells and others. TRPV2 modulates various cellular functions in these cells. In addition to the expression of TRPV2 in normal tissues, TRPV2 is expressed in various types of tumor cells—in bladder tumors, prostate cancer cells, hepatocellular carcinoma, hepatoma cell line, gliomas and glioblastoma [23]. There is some evidence that effective blockage of TRPV2 channels may offer an innovative means of inhibiting cancer metastases [22]. As was mentioned, despite the unique function and regulation in cells of various tissues and organs, the physiological role of TRPV2 has not been completely elucidated. The topical task is to assess the role of the TRPV2 channel in many biological processes carefully using multiple approaches. In the absence of more specific antagonists, marine cyclic guanidine alkaloids monanchomycalin B (**1**) and urupocidin A (**2**) may find applications in the study of TRPV2.

It was shown earlier that monanchomycalin B (**1**) exhibited potent cytotoxic activities against HL-60 human leukemia cells [5] which may be partially explained by the inhibition of TRP channels (although the issue of cytotoxicity and its mechanism require more detailed study). TRP channels have been shown to play a role in many cancer types and the TRP channel–mediated disruption of Ca^2+^ homeostasis triggers important cancer cell phenotype changes [17,22]. Thus, natural products, their derivatives and TRP-directed drug delivery constructs may yield an original means of tackling cancers with potential applications in medicine, e.g., for the selective pharmaco-delivery of cytotoxic payloads to diseased tissues, providing an innovative platform in chemical biology and molecular medicine.

## 3. Materials and Methods

### 3.1. General

The ^1^H- and ^13^C-NMR spectra were recorded on Bruker DRX-500 and Avance III-700 spectrometers at 500, 700, and 125, and 175 MHz, respectively, with Me_4_Si as an internal standard. ESI mass spectra (including HR ESI-MS) were obtained on an Agilent 6510 Q-TOF LC-MS spectrometer (Agilent Technologies, Santa Clara, CA, USA) by direct infusion in MeOH. Low-pressure column liquid chromatography was performed using Sephadex LH-20 (Sigma-Aldrich Co., St. Louis, MO USA). Si gel plates (4.5 × 6.0 cm, 5–17 µm, Sorbfil, Moscow, Russia) were used for thin-layer chromatography and compounds were visualized by spraying with 10% H_2_SO_4_ solution followed by heating. The flash column chromatography was carried out using microcolumn with YMC*GEL ODS-A (75 µm). HPLC was performed using an Agilent Series 1100 (Agilent Technologies, Santa Clara, CA USA) and Shimadzu (Shimadzu Corporation, Kyoto, Japan) instruments equipped with the refractive index detector RID-DE14901810 and an YMC-ODS-A (250 × 10 mm) column (YMC CO., LTD., Kyoto, Japan).

### 3.2. Animal Material

The sponge *M. pulchra* (Lambe, 1894) was collected by dredging during a scientific cruises onboard the R/V “Academic Oparin”, the sample N 047-243: August 2015, Onekotan Island from a depth of approximately 138 m (49°24′6′′ N; 154°17′48′′ E,), the sample N 043-583: August 2012, Urup Island from a depth of approximately 207 m (45°55′6′′ N; 143°41′36′′ E,) and identified by Krasokhin V. B. The voucher specimens are kept in the collection of PIBOC.

### 3.3. Extraction and Isolation

***Monanchomycalin B* (1):** The fresh collection of the sample *M. pulchra* (N 047-243) was extracted with EtOH and a part of which (30 mL) was concentrated in vacuo. The residue was chromatographed over a microcolumn (10 × 12 mm) with YMC*Gel ODS-A reversed-phase sorbent (75 µm) using aqueous EtOH (40%) and then EtOH (65%)–H_2_O (35%)–TFA (0.1%). The eluates with TFA were evaporated. The compounds were isolated by HPLC using YMC-ODS-A column (250 × 10 mm) and EtOH (65%)–H_2_O (35%)–TFA (0.1%) to afford pure compound **1** (4.0 mg), RT = 18.5 min; HRMALDI-TOF-MS *m*/*z* 785.6288 [M + H]^+^, HRESI MS *m*/*z* 785.6259 [M + H]^+^ (calcd. for C_45_H_81_N_6_O_5_: 785.6263); 393.3160 [M + 2H]^2+^ (calcd. for C_45_H_82_N_6_O_5_/2: 393.3168).

***Urupocidin A* (2):** The fresh collection of the sample *M. pulchra* (N 043-583) was immediately frozen and kept at −20 °C. The biological material (dry weight 10.5 g) was extracted with EtOH (200 mL × 3). The combined EtOH extract was concentrated and partitioned between *n*-BuOH and H_2_O. The butanol layer was separated and concentrated in vacuo. The butanol-soluble materials were further partitioned between *n*-hexane and aqueous EtOH. The aqueous EtOH-soluble materials were concentrated and further separated by CC on Sephadex LH-20 (elution with EtOH) to obtain a mixture of guanidine alkaloids, which were subjected to preparative HPLC (YMC-ODS-A column (250 × 10 mm), 65:35:0.1% EtOH–H_2_O–TFA) to provide 15.5 mg of urupocidin A (**2**) (0.14%, yield, dry wt.). RT = 15.0 min; HRESIMS *m*/*z* 547.3956 [M + H]^+^ (calcd. for C_29_H_50_N_6_O_4_ 547.3966).

***Hydrogenation of Urupocidin A* (2):** Experiments were performed as previously described [8]. In brief, PtO_2_ was added to a solution of urupocidin A (3.1 mg) in MeOH (1.0 mL) and stirred under H_2_ at 25 °C for 1 h. The catalyst was removed by filtration and the solvent by evaporation to give a residue, which was then washed off from the impurities by cold chloroform. The result was obtained 1.3 mg of hydrogenated derivative of the urupocidin A (**3**). HRESIMS *m*/*z* 551.4279 [M + H]^+^ (calcd. for C_29_H_54_N_6_O_4_ 551.4298).

***Methanolysis of Urupocidin A* (2):** Experiments were performed as previously described [8]. In brief, a mixture of 0.1 N NaOMe (380 µL) in MeOH (940 µL) and urupocidin A (4.5 mg) was heated at 65 °C for 17 h. The solvent was removed under Ar and the residue dissolved in H_2_O (1 mL), neutralized with dilute CH_3_COOH, and extracted with CHCl_3_ (3 × 1 mL). The CHCl_3_ extracts were evaporated in vacuo to give a brown oil, which was separated by preparative HPLC (YMC-ODS-A column (250 × 10 mm), 70% EtOH/0.1% TFA and then 75% EtOH/0.1% TFA) to give the pure compound **4** (0.9 mg). RT = 18.5 min; HRESIMS *m/z* 432.3229 [M + H]^+^ (calcd. for C_25_H_41_N_3_O_3_ 432.3221).

### 3.4. Inhibitory Activities on TRPV1, TRPV2, TRPV3 and TRPA1

The mouse TRPV2 gene was cloned form pancreatic beta cell line MIN6 and the CHO cell line stably expressing mouse TRPV2 was generated using T-Rex System (Thermo Fisher Scientific Inc., Waltham, MA, USA) similarly previously obtained stable cell lines for rat TRPV1, human TRPV3 and rat TRPA1 [6,8]. Fluorescent assays were performed as described in [6]. The alkaloid’s solutions of desired concentration were obtained by serial dilution of water stock solutions (10 mg/mL) in calcium assay buffer (10 mM HEPES/HBSS, pH 7.4). The buffer alone (negative control) or serial dilutions of alkaloids **1**–**4** (20 μl) were added to the cells loaded with the cytoplasmic calcium indicator Fluo-4AM using Fluo-4 Direct™ Calcium Assay Kit (Thermo Fisher Scientific Inc., Waltham, MA, USA) (80 μl) and the cells were incubated 30 min at 37 °C. Changes in cell fluorescence (λ_ex_ = 485 nm, λ_em_ = 520 nm) were monitored using NOVO star (BMG LABTECH, Ortenberg, Germany) before and after the addition of relevant agonist (500 nM capsaicin for rTRPV1, 300 μM 2APB for mTRPV2, 200 μM 2APB for hTRPV3, 100 μM AITC for rTRPA1) at 25 °C. Average cell responses to agonists in controls were about 60, 45, 40, 85% of the responses produced by 5 μM ionomycin for rTRPV1, mTRPV2, hTRPV3, rTRPA1 correspondingly. To avoid the spread of the data due to prolonged instrumental measurement each experimental point has control in the nearest plate wells. Curve fitting and parameter estimations were performed with Microsoft Excel 2007 (Microsoft Corporation, Redmond, WA, USA) and Origin 7.0 (OriginLab Corporation, Northampton, MA, USA). The EC_50_ values were determined as the concentration of test substance required to produce half-maximal decreases in [Ca^2+^].

## 4. Conclusions

Two guanidine natural products, monanchomycalin B (**1**) and urupocidin A (**2**), have been isolated from new collections of the marine sponge *M. pulchra*. Two semi-synthetic urupocidin A derivatives were obtained by chemical modifications of **2**. The guanidine alkaloids **1**–**3** are capable of interacting with rTRPV1, mTRPV2 and hTRPV3 receptors and **1** is the most active. All compounds **1**–**4** were not active against the rat TRPA1 channel. Characterized new TRPV(1–3) antagonists could provide interesting molecular tools in the search for new pharmacological agents and for developing a new generation of therapeutics to assess the role of TRPV channels in different cells including the pathobiology of cancer cells.

## Figures and Tables

**Table 1:** Inhibitory activity of compounds **1**–**4** against rTRPV1, mTRPV2, hTRPV3 and rTRPA1.

Compounds	EC_50_ (µM)			
	rTRPV1	mTRPV2	hTRPV3	rTRPA1
**1**	6.02 ± 0.36	2.84 ± 1.01	3.25 ± 0.60	not active
**2**	21.47 ± 2.51	28.06 ± 6.65	23.55 ± 2.03	not active
**3**	52.82 ± 5.31	nd ^1^	29.19 ± 3.18	not active
**4**	435.07 ± 49.92	nd ^1^	193.33 ± 25.47	not active

^1^ Not detected, due to small amount of compounds.

## References

[1] Blunt J.W., Copp B.R., Keyzers R.A., Munro M.H.G., Prinsep M.R. (2016). Marine natural products. Nat. Prod. Rep..

[2] Berlinck R.G.S., Romminger S. (2016). The chemistry and biology of guanidine natural products. Nat. Prod. Rep..

[3] Guzii A.G., Makarieva T.N., Denisenko V.A., Dmitrenok P.S., Kuzmich A.S., Dyshlovoy S.A., Krasokhin V.B., Stonik V.A. (2010). Monanchocidin: A new apoptosis-inducing polycyclic guanidine alkaloid from the marine sponge *Monanchora pulchra*. Org. Lett..

[4] Makarieva T.N., Tabakmaher K.M., Guzii A.G., Denisenko V.A., Dmitrenok P.S., Shubina L.K., Kuzmich A.S., Lee H.S., Stonik V.A. (2011). Monanchocidins B-E: Polycyclic guanidine alkaloids with potent antileukemic activities from the sponge *Monanchora pulchra*. J. Nat. Prod..

[5] Makarieva T.N., Tabakmaher K.M., Guzii A.G., Denisenko V.A., Dmitrenok P.S., Kuzmich A.S., Lee H.S., Stonik V.A. (2012). Monanchomycalins A and B, unusual guanidine alkaloids from the sponge *Monanchora pulchra*. Tetrahedron Lett..

[6] Tabakmaher K.M., Denisenko V.A., Guzii A.G., Dmitrenok P.S., Lee H.S., Makarieva T.N. (2013). Monanchomycalin C, a new pentacyclic guanidine alkaloid from the Far-Eastern marine sponge *Monanchora pulchra*. Nat. Prod. Commun..

[7] Tabakmakher K.M., Makarieva T.N., Denisenko V.A., Guzii A.G., Dmitrenok P.S., Kuzmich A.S., Stonik V.A. (2015). Normonanchocidins A, B and D, new pentacyclic guanidine alkaloids from the Far-Eastern marine sponge *Monanchora pulchra*. Nat. Prod. Commun..

[8] Makarieva T.N., Ogurtsova E.K., Denisenko V.A., Dmitrenok P.S., Tabakmakher K.M., Guzii A.G., Pislyagin E.A., Es’kov A.A., Kozhemyako V.B., Aminin D.L. (2014). Urupocidin A: A new, inducing iNOS expression bicyclic guanidine alkaloid from the marine sponge *Monanchora pulchra*. Org. Lett..

[9] Guzii A.G., Makarieva T.N., Korolkova Y.V., Andreev Y.A., Mosharova I.V., Tabakmaher K.M., Denisenko V.A., Dmitrenok P.S., Ogurtsova E.K., Antonov A.S. (2013). Pulchranin A, isolated from the Far-Eastern marine sponge, *Monanchora pulchra*: The first marine non-peptide inhibitor of TRPV-1 channels. Tetrahedron Lett..

[10] Makarieva T.N., Ogurtsova E.K., Korolkova Y.V., Andreev Y.A., Mosharova I.V., Tabakmaher K.M., Guzii A.G., Denisenko V.A., Dmitrenok P.S., Lee H.S. (2013). Pulchranins B and C, new acyclic guanidine alkaloids from the Far-Eastern marine sponge *Monanchora pulchra*. Nat. Prod. Commun..

[11] Dyshlovoy S.A., Hauschild J., Amann K., Tabakmakher K.M., Venz S., Walther R., Guzii A.G., Makarieva T.N., Shubina L.K., Fedorov S.N. (2015). Marine alkaloid Monanchocidin A overcomes drug resistance by induction of autophagy and lysosomal membrane permeabilization. Oncotarget.

[12] Ogurtsova E.K., Makarieva T.N., Korolkova Y.V., Andreev Y.A., Mosharova I.V., Denisenko V.A., Dmitrenok P.S., Lee Y.J., Grishin E.V., Stonik V.A. (2015). New derivatives of natural acyclic guanidine alkaloids with TRPV receptor-regulating properties. Nat. Prod. Commun..

[13] Kudryavtsev D., Makarieva T., Utkina N., Santalova E., Kryukova E., Methfessel C., Tsetlin V., Stonik V., Kasheverov I. (2014). Marine natural products acting on the acetylcholine-binding protein and nicotinic receptors: From computer modeling to binding studies and electrophysiology. Mar. Drugs.

[14] Gees M., Owsianik G., Nilius B., Voets T. (2012). TRP channels. Compr. Physiol..

[15] Smani T., Shapovalov G., Skryma R., Prevarskaya N., Rosado J.A. (2015). Functional and physiopathological implications of TRP channels. Biochim. Biophys. Acta.

[16] Nilius B., Szallasi A. (2014). Transient receptor potential channels as drug targets: From the science of basic research to the art of medicine. Pharmacol. Rev..

[17] Kaneko Y., Szallasi A. (2014). Transient receptor potential (TRP) channels: A clinical perspective. Br. J. Pharmacol..

[18] Mickle A.D., Shepherd A.J., Mohapatra D.P. (2015). Sensory TRP channels: The key transducers of nociception and pain. Prog. Mol. Biol. Transl. Sci..

[19] Brederson J.D., Kym P.R., Szallasi A. (2013). Targeting TRP channels for pain relief. Eur. J. Pharmacol..

[20] Colsoul B., Nilius B., Vennekens R. (2013). Transient receptor potential (TRP) cation channels in diabetes. Curr. Top. Med. Chem..

[21] De Logu F., Patacchini R., Fontana G., Geppetti P. (2016). TRP functions in the broncho-pulmonary system. Semin. Immunopathol..

[22] Gautier M., Dhennin-Duthille I., Ay A.S., Rybarczyk P., Korichneva I., Ouadid-Ahidouch H. (2014). New insights into pharmacological tools to TR(i)P cancer up. Br. J. Pharmacol..

[23] Kojima I., Nagasawa M. (2014). TRPV2. Handb. Exp. Pharmacol..

[24] Appendino G., Minassi A., Pagani A., Ech-Chadad A. (2008). The role of natural products in the ligand deorphanization of TRP channels. Curr. Pharm. Des..

[25] Kashman Y., Hirsh S., McConnell O.J., Ohtani I., Kusumi T., Kakisawa H. (1989). Ptilomycalin A: A novel polycyclic guanidine alkaloid of marine origin. J. Am. Chem. Soc..

[26] Heding A., Elling C.E., Schwartz T.W. (2002). Novel method for the study of receptor Ca^2+^ signalling exemplified by the NK1 receptor. J. Recept. Signal Transduct. Res..

[27] Ogurtsova E., Makarieva T., Guzii A., Dmitrenok P., Denisenko V., Krasokhin V., Korolkova Y., Andreev Y., Mosharova I., Grishin E. (2015). Inhibitory Activity on TRP Receptors of Pentacyclic Alkaloids from the Sponge *Haliclona (Gellius)* sp.. Chem. Nat. Compd..

[28] Cordero-Morales J.F., Gracheva E.O., Julius D. (2011). Cytoplasmic ankyrin repeats of transient receptor potential A1 (TRPA1) dictate sensitivity to thermal and chemical stimuli. Proc. Natl. Acad. Sci. USA.

[29] Colton C.K., Zhu M.X. (2007). 2-Aminoethoxydiphenyl borate as a common activator of TRPV1, TRPV2, and TRPV3 channels. Handb. Exp. Pharmacol..

[30] Alpizar Y.A., Boonen B., Gees M., Sanchez A., Nilius B., Voets T., Talavera K. (2014). Allyl isothiocyanate sensitizes TRPV1 to heat stimulation. Pflugers Arch..

[31] Koizumi K., Iwasaki Y., Narukawa M., Iitsuka Y., Fukao T., Seki T., Ariga T., Watanabe T. (2009). Diallyl sulfides in garlic activate both TRPA1 and TRPV1. Biochem. Biophys. Res. Commun..

[32] Leffler A., Lattrell A., Kronewald S., Niedermirtl F., Nau C. (2011). Activation of TRPA1 by membrane permeable local anesthetics. Mol. Pain.

[33] Mihara S., Shibamoto T. (2015). The role of flavor and fragrance chemicals in TRPA1 (transient receptor potential cation channel, member A1) activity associated with allergies. Allergy Asthma Clin. Immunol..

[34] Chen J., Zhang X.F., Kort M.E., Huth J.R., Sun C., Miesbauer L.J., Cassar S.C., Neelands T., Scott V.E., Moreland R.B. (2008). Molecular determinants of species-specific activation or blockade of TRPA1 channels. J. Neurosci..

